# The role of STK11/LKB1 in cancer biology: implications for ovarian tumorigenesis and progression

**DOI:** 10.3389/fcell.2024.1449543

**Published:** 2024-10-31

**Authors:** Jian Kang, Stefano Gallucci, Junqi Pan, Jonathan S. Oakhill, Elaine Sanij

**Affiliations:** ^1^ St Vincent’s Institute of Medical Research, Melbourne, VIC, Australia; ^2^ Department of Medicine-St Vincent’s Hospital, University of Melbourne, Melbourne, VIC, Australia; ^3^ Department of Biochemistry and Molecular Biology, Monash University, Melbourne, VIC, Australia; ^4^ Division of Cancer Research, Peter MacCallum Cancer Centre, Melbourne, VIC, Australia

**Keywords:** LKB1, STK11, cancer biology, ovarian cancer, metabolism, immunotherapy, targeted therapy, cancer signaling

## Abstract

STK11 (serine-threonine kinase 11), also known as LKB1 (liver kinase B1) is a highly conserved master kinase that regulates cellular metabolism and polarity through a complex signaling network involving AMPK and 12 other AMPK-related kinases. Germline mutations in *LKB1* have been causatively linked to Peutz-Jeghers Syndrome (PJS), an autosomal dominant hereditary disease with high cancer susceptibility. The identification of inactivating somatic mutations in *LKB1* in different types of cancer further supports its tumor suppressive role. Deleterious mutations in *LKB1* are frequently observed in patients with epithelial ovarian cancer. However, its inconsistent effects on tumorigenesis and cancer progression suggest that its functional impact is genetic context-dependent, requiring cooperation with other oncogenic lesions. In this review, we summarize the pleiotropic functions of LKB1 and how its altered activity in cancer cells is linked to oncogenic proliferation and growth, metastasis, metabolic reprogramming, genomic instability, and immune modulation. We also review the current mechanistic understandings of this master kinase as well as therapeutic implications with particular focus on the effects of LKB1 deficiency in ovarian cancer pathogenesis. Lastly, we discuss whether LKB1 deficiency can be exploited as an Achilles heel in ovarian cancer.

## Introduction

The gene *STK11* (serine-threonine kinase 11), also known as *LKB1* (liver kinase B1) encodes a highly conserved serine threonine kinase. The full-length human LKB1 protein encompasses three domains including an N-terminal domain with nuclear localization signal, a central domain with kinase catalytic activity and a C-terminal domain with regulatory function (Hezel and Bardeesy, 2008). LKB1 is a constitutively active master kinase that regulates cellular metabolism and polarity through a complex signaling network. LKB1 forms a heterotrimeric complex with a pseudokinase STRAD (STE20-related adaptor) and a scaffolding protein CAB39 (calcium-binding protein 39, as known as MO25) (Baas et al., 2003; Boudeau et al., 2003). This interaction leads to its translocation from the nucleus to the cytoplasm where LKB1 phosphorylates AMPK (AMP-activated protein kinase) and 12 other AMPK-related kinases including MARK-1/2/3/4 (microtubule-associated protein/microtubule affinity regulating kinases-1/2/3/4), NUAK1/2 (NUA kinase family 1/2), SIK-1/2/3 (salt-inducible kinase-1/2/3), BRSK-1/2 (brain-specific kinase-1/2), and SNAK (sucrose non-fermenting protein-related kinase) (Jaleel et al., 2005; Lizcano et al., 2004). A recent study identified that LKB1 also phosphorylates and activates TSSK1B (testis-specific serine/threonine-protein kinase 1B), a member of the calcium/calmodulin-dependent protein kinase superfamily (Kim et al., 2024).

The signaling pathway through AMPK is one of the best characterized pathways downstream of LKB1. AMPK is an evolutionarily conserved protein in eukaryotic cells, composed of 3 subunits. The α subunit contains the catalytically active kinase domain while the β and γ subunits serve regulatory and localization roles. AMPK is a sensor of cellular energy homeostasis and can be activated by the increased ratios of AMP to ATP and ADP to ATP due to energy stresses (*e.g.*, glucose restriction or hypoxia). LKB1 in complex with STRAD and CAB39, activates AMPK by phosphorylating Threonine 172 in the T-loop of the α kinase domain (Hawley et al., 2003). This leads to suppression of anabolic processes (e.g., synthesis of proteins, fatty acids and glycogen) and stimulation of catabolic processes (e.g., fatty acid oxidation, glycolysis and autophagy) to restore cell energy balance and regulate cell proliferation and growth (Gwinn et al., 2008; Shaw, 2009; Shaw et al., 2004). Moreover, the LKB1-AMPK pathway regulates epithelial tight junction assembly and cellular polarity by remodeling the actin cytoskeleton (Zhang et al., 2006; Zheng and Cantley, 2007).

Similarly, LKB1 can phosphorylate the T-loop of the catalytic subunit of 12 other AMPK-related kinases and increase their activities (Lizcano et al., 2004). These kinases mainly regulate cellular polarity and metabolism (Bright et al., 2008; Jaleel et al., 2005; Sun et al., 2020; Thirugnanam and Ramchandran, 2020; Timm et al., 2003; van de Vis et al., 2021). Unlike well-characterized LKB1-AMPK signaling pathway, how the biological effects of LKB1 are mediated by these kinases are less studied. Nevertheless, accumulating evidence supports that they are the major mediators of the functional impacts of LKB1 in cancer pathogenesis. In this review, we summarize the pleiotropic roles of LKB1 in cancer biology including oncogenic proliferation and growth, metastasis, metabolic reprogramming, genomic instability, and immune modulation. We particularly review the effects of LKB1 deficiency in pathogenesis of ovarian cancer and discuss whether LKB1 deficiency could be exploited as an Achilles heel in ovarian cancer.

## *LKB1* mutations in cancers

Germline mutations in *LKB1* have been causatively linked to Peutz-Jeghers Syndrome (PJS), an autosomal dominant hereditary disease (Hemminki et al., 1998; Jenne et al., 1998). This disease is characterized by the formation of hamartomatous polyps in the gastrointestinal tract, mucocutaneous pigmentation, and high cancer susceptibility with more than 93% of patients developing malignancies by an average age of 43 years. These germline mutations eliminate the kinase activity of LKB1 (Hemminki et al., 1998; Jenne et al., 1998; Mehenni et al., 1998). The identification of inactivating somatic mutations in LKB1 in different types of cancer further supports its tumor suppressor role.

**FIGURE 1:**
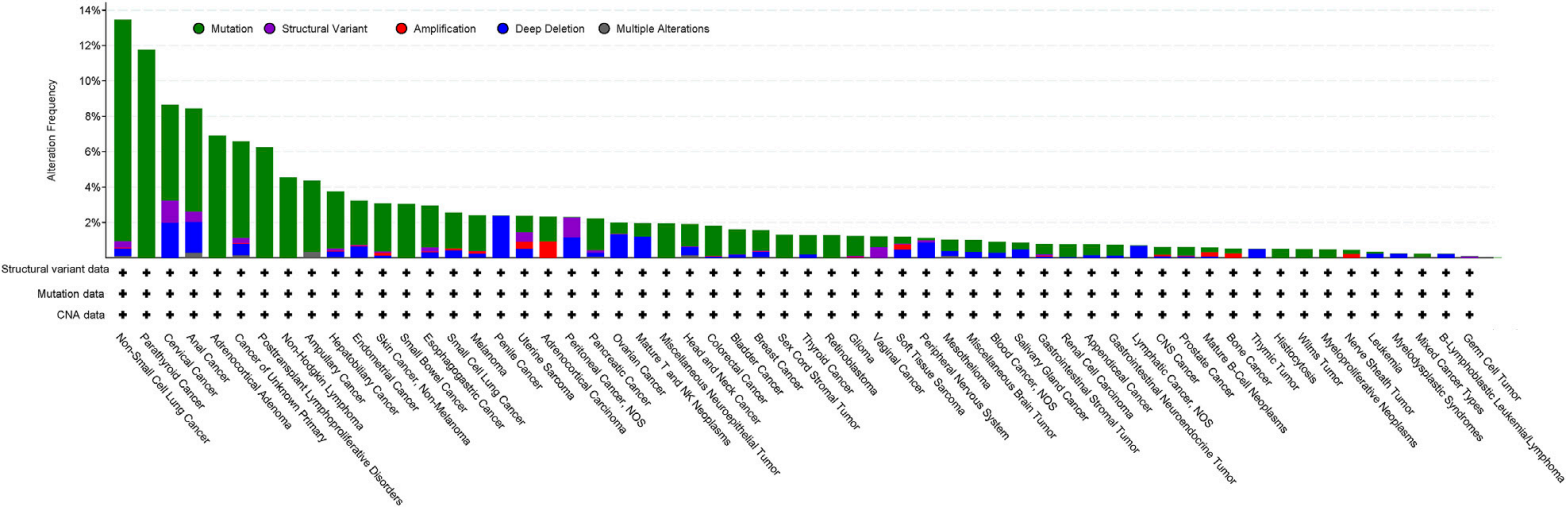
Analysis of the frequency of *STK11*/*LKB1* mutations in cancer using the AACR project GENIE data set (v15.1) via cBioPortal (http://cbioportal.org) (access date 5th June 2024).

Analysis of the AACR project GENIE data set (v15.1) via cBioPortal shows genetic alterations of *LKB1* in 3% of 160,994 cancer samples, of which the greatest prevalence of alterations is observed in non-small cell lung cancer (NSCLC) (13.46%), parathyroid cancer (11.76%), cervical cancer (8.64%), anal cancer (8.43%) and adrenocortical adenoma (6.9%) (Figure 1). The most common somatic mutations in *LKB1* are missense mutations (2.07%), truncating mutations (0.21%), splice mutations (0.52%) and fusion mutations (0.21%). Notably, 30% of the mutations in NSCLC are missense mutations which disrupt its catalytic function and are predicted to be oncogenic (Gill et al., 2011; Granado-Martínez et al., 2020).

Beyond genetic mutations, LKB1 expression can be regulated by epigenetic mechanisms including promoter hypermethylation or small non-coding RNAs by targeting *LKB1* 3′UTR region and impairing *LKB1* mRNA translation (Borzi et al., 2020). Posttranslational modifications of LKB1 including phosphorylation, ubiquitination, SUMOylation have been also reported to impact its protein abundance and function by regulation of intracellular localization, protein stability, conformation, and protein-protein interactions (Hu et al., 2023). Two recent reviews provide extensive overview of epigenetic and post-translational regulation of LKB1 expression (Borzi et al., 2020; Hu et al., 2023).

## Functional impacts of LKB1 mutations in cancer pathogenesis

### Tumorigenesis

The role of LKB1 in oncogenic transformation has been meticulously studied in mouse models. Homozygous loss of *Lkb1* leads to mouse embryonic death, whereas mice with *Lkb1* heterozygous deletion developed intestinal polyposis that recapitulate the histological changes found in patients with PJS (Bardeesy et al., 2002). Furthermore, mice with germline *Lkb1* haploinsufficiency developed gastric harmartomas after 20 weeks of age and hepatocellular carcinoma after 30 weeks (Miyoshi et al., 2002; Nakau et al., 2002).

Somatic homozygous inactivation of *Lkb1* in mouse endometria was found to promote the development of aggressive endometrial cancer much more potently than heterozygous endometrial *Lkb1* inactivation (Contreras et al., 2010). A similar finding was reported in a genetically engineered mouse model of pancreatic cancer (Morton et al., 2010). However, more studies reported contradictory findings in which genetic manipulation of *Lkb1* in mouse tissues did not promote prostate cancer (Hermanova et al., 2020), bladder cancer (Shorning et al., 2011), serous ovarian cancer (Tanwar et al., 2014), lung cancer (Ji et al., 2007), melanoma (Liu et al., 2012) or endometrial cancer (Cheng et al., 2014). While these findings suggest a tissue-specific role of LKB1 in tumorigenesis, increasing evidence from mouse models shows that LKB1 inactivation cooperates with other oncogenic events to promote tumorigenesis (Li and Zhu, 2020; Skoulidis and Heymach, 2019). Concurrent loss of *Lkb1* and *Tp53* inactivation promotes the development of osteosarcoma, lymphoma, sarcoma (Wei et al., 2005) and hepatic carcinoma *in vivo* (Takeda et al., 2006). Inactivation of LKB1 in mice carrying oncogenic *Kras* mutations leads to development of intraductal papillary mucinous neoplasm (a precursor of pancreatic cancer) (Collet et al., 2020), pancreatic cancer (Morton et al., 2010), lung adenocarcinoma (Ji et al., 2007; Murray et al., 2022; Rogers et al., 2017) and melanoma (Liu et al., 2012). Mice with concurrent loss of *Pten* and *Lkb1* develop endometrial cancer (Cheng et al., 2014) bladder carcinoma (Shorning et al., 2011), lung squamous cell carcinoma (Xu et al., 2014), ovarian carcinoma (Tanwar et al., 2014), and prostate cancer (Hermanova et al., 2020). These findings suggest that LKB1 loss might occur as a secondary event that facilitate the transformation of oncogene-driven cancers.

### Tumor growth and metastasis

**FIGURE 2:**
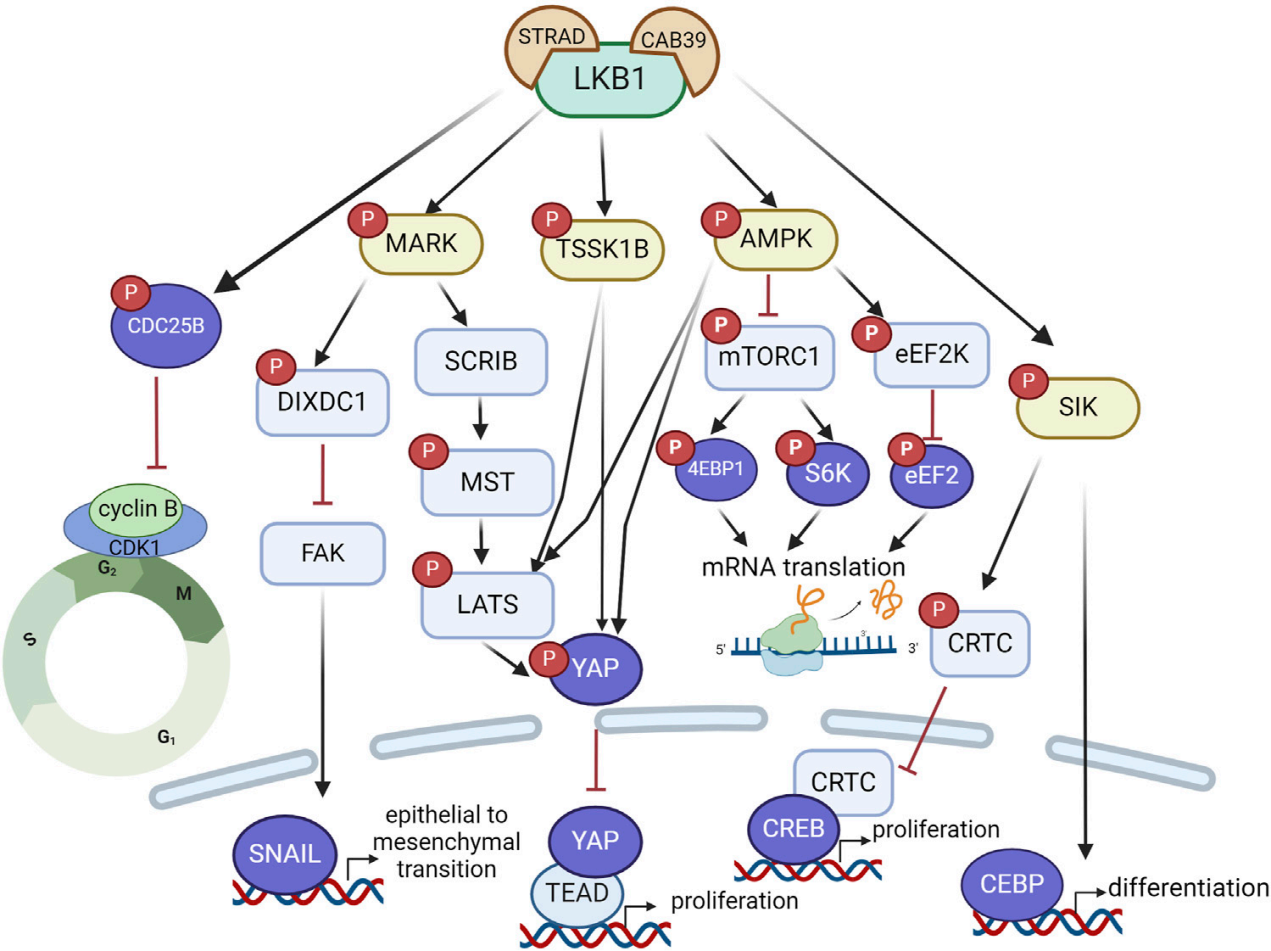
LKB1 functions as a tumor suppressor to restrain tumor growth and metastasis via a complex signaling network that regulate mRNA translation, protein synthesis, and transcriptional programs associated with cell proliferation, differentiation and epithelial mesenchymal transition. Created with BioRender.com.

As a tumor suppressor, LKB1 inactivation not only contributes to oncogenic transformation but also promotes tumor growth mainly through mTORC1-dependent and independent regulation of mRNA translation and growth control (Figure 2). LKB1 negatively regulates mTORC1 through phosphorylation of the mTORC1 upstream regulator TSC2 (TSC complex subunit 2) and the mTORC1 complex component RAPTOR (regulatory associated protein of MTOR complex 1) by AMPK. 4EBP1 (Eukaryotic translation initiation factor 4E-binding protein 1) and S6K1 (ribosomal S6 kinase) are the main effectors of mTORC1-dependent regulation of mRNA translation and growth control (Shackelford and Shaw, 2009). Studies in mouse models of invasive endometrial tumors show that loss of *Lkb1* or concurrent loss of *Pten* and *Lkb1* confer a strong dependence on mTOR signaling for tumor growth, rendering them hypersensitive to mTOR inhibition (Cheng et al., 2014; Contreras et al., 2010). This sensitivity was also observed in lung cancer cell lines with *LKB1* and *KRAS* co-mutations, but the single mutation did not confer it. The mTOR inhibitor everolimus, has shown promising activity and safety profile in a phase II clinical trial (NCT02352844) in patients with solid tumors harboring hyperactive mTORC1 signaling due to *TSC1/2*, *NF1/2* or *LKB1* mutations (Table 1) (Devarakonda et al., 2021). Notably, one patient with *LKB1* mutated advanced lung adenocarcinoma showed stable disease after receiving 10 mg of everolimus orally for 28 days, supporting the potential clinical utility of everolimus for treating LKB1 mutant tumors. AMPK also regulates mRNA translation through mTORC1-independent eEF2K (eukaryotic translation elongation factor 2 kinase) – eEF2 (eukaryotic translation elongation factor 2) pathway. eEF2 is a translation factor necessary for peptide translocation during the elongation phase of protein synthesis. Under metabolic stress, the energy sensor AMPK activates eEF2K which negatively regulates eEF2 activity through phosphorylation and thus reduces protein synthesis to preserve energy (Johanns et al., 2017).

**TABLE 1:** The clinical trials in patients with LKB1-mutated tumors.

NCT #	Drug	Study design	Study result	Study phase	Status
NCT02352844 (Devarakonda et al., 2021)	everolimus	This study evaluates the response to everolimus, a mTORC1 inhibitor in advanced solid tumors with *TSC1*, *TSC2*, *NF1*, *NF2*, or *LKB1* mutations	Of 8 patients who could be evaluated, one patient experienced complete response and another experienced stable disease. The patient with stable disease had lung adenocarcinoma with LKB1 mutation	II	Completed
NCT03600883 (Skoulidis et al., 2021)	sotorasib	This study evaluates the safety, tolerability, pharmacokinetics, pharmacodynamics, and efficacy of sotorasib (AMG 510), a KRAS G12C inhibitor, in advanced solid tumors with KRAS G12C mutation, in monotherapy and combination therapy. *LKB1* mutation was assessed as co-occurring genomic alteration with *KRAS* G12C	Of the 124 patients with NSCLC who could be evaluated, 46 patients (37.1%) I/II showed an objective response, including 4 patients (3.2%) having a complete response and 42 (33.9%) having a partial response. The median progressionfree survival was 6.8 months, and the median overall survival was 12.5 months. Among the 104 patients who were assessed for co-occurring genomic alterations, a response was seen in 50% of the patients in the subgroup with mutated LKB1 and wild-type KEAP1. Among patients with mutated KEAP1, a response was seen in 23% of those with both mutated LKB1 and KEAP1 and in 14% of those with wild-type LKB1 and mutated KEAP1	I/II	active, not recruiting
NCT04933695 (ClinicalTrials, 2021)	sotorasib	This study evaluates the clinical activity of sotorasib (AMG 510), a *KRAS* G12C inhibitor in stage IV NSCLC tumors with *KRAS* G12C mutation and <1% PD-L1 and/or *LKB1* co-mutation in need of first line treatment	N/A	II	active, not recruiting
NCT03785249 (Pant et al., 2023)	MRTX849	This study evaluates the safety, tolerability, pharmacokinetics, pharmacodynamics, and clinical activity of MRTX849 (adagrasib), a *KRAS* G12C inhibitor, in advanced solid tumors with a *KRAS* G12C mutation. Advanced solid tumor malignancies with *KRAS* G12C mutation stratified by co-mutation status (e.g., LKB1)	Of 51 patients with *KRAS* G12C–mutant advanced NSCLC, a partial response rate was 45% (23/51) and 51% had stable disease (26/51). The objective response rate in patients with *LKB1* co-mutations was 64% (9/14). Patients with *LKB1* co-mutations showed increase of CD4 and CD8 expression after treatment, indicating a potential immune response to therapy	I/II	recruiting
NCT05276726 (ClinicalTrials, 2022)	Glecirasib	This study evaluates the safety, tolerability and preliminary antitumor activity of Glecirasib (JAB-21822), a *KRAS* G12C inhibitor, in locally advanced or metastatic NSCLC with concurrent *KRAS* G12C and *LKB1* mutation and KEAP wild type either treatment naive or at least one-line prior therapy for advance disease	N/A	Ib/II	recruiting
NCT03872427 (ClinicalTrials, 2019)	telaglenastat	This study evaluates the clinical activity of telaglenastat (CB-839), a glutaminase inhibitor, in solid tumors or malignant peripheral nerve sheath tumors with *NF1*, *KEAP1*/*NRF2*, or *LKB1* mutations	N/A	II	active, not recruiting
NCT06188208 (ClinicalTrials, 2023b)	VVD-130850	This study evaluates the safety, tolerability, pharmacokinetics, pharmacodynamics, and anti-tumor activity of VVD-130850, a STAT3 Inhibitor, as monotherapy and in combination with immunotherapy in advanced solid and hematologic tumors. LKB1 mutated NSCLC was recruited for combination therapy	N/A	I	recruiting
NCT05807048 (ClinicalTrials, 2023a)	daratumumab	This study evaluates the clinical activity of daratumumab, an anti-PD-L1 monoclonal antibody in metastatic NSCLC with *LKB1* mutation who have received previous standard treatment. An overall response rate ≥20% is considered clinically meaningful	N/A	II	recruiting
NCT04265534 (ClinicalTrials, 2020)	telaglenastat with pembrolizumab and chemotherapy	This study evaluates the safety and clinical activity of telaglenastat (CB-839), a glutamase inhibitor, with pembrolizumab and chemotherapy, for first line treatment of metastatic NSCLC with *NF1, KEAP1/NRF2, or LKB1* mutations	Lack of clinical benefit and terminated	II	Terminated
NCT04173507 (Skoulidis et al., 2022)	talazoparib plus avelumab	This study (a LUNG-MAP treatment trial) evaluates the efficacy and safety of talazoparib, a PARP inhibitor in combination with avelumab, a PD-L1 monoclonal antibody, for stage IV or recurrent non-squamous NSCLC with *LKB1* mutations	Of 42 patients, the objective response rate was 2% (1/42), the disease control rate at 12 weeks was 40% (17/42). 62% patients (26/42) had stable disease as best objective response. The median progression free survival was 2.7 months, and the median overall survival was 7.6 months. However, the combination therapy did not meet the pre-specified threshold for efficacy	II	Completed
NCT05887492 (ClinicalTrials, 2023c)	TNG260 plus pembrolizumab	This study evaluates the safety and tolerability, pharmacokinetics, and clinical activity of TNG260, a CoREST-selective deacetylase inhibitor, in combination with pembrolizumab, a PD-1 monoclonal antibody, in locally advanced or metastatic *LKB1* mutated solid tumors	N/A	I/II	recruiting
NCT06124963 (ClinicalTrials, 2023d)	WX390 plus toripalimab	This study evaluates the safety, tolerability and preliminary antitumor activity of WX390, a PI3K-mTOR dual inhibitor, in combination with toripalimab, a PD-1 monoclonal antibody in advanced gastric-type endocervical adenocarcinoma with *LKB1* mutations	N/A	II	recruiting
NCT03184571 (ClinicalTrials, 2017)	bemcentinib plus pembrolizumab	This study evaluates the safety and anti-tumor activity of bemcentinib, an AXK inhibitor, in combination with pembrolizumab, a PD-1 monoclonal antibody in advanced NSCLC, some of which have *LKB1* mutations	Of the 24 chemo-refractory and 30 PD-1 inhibitor refractory NSCLC, three patients had LKB1 mutations and all experienced clinical benefit. One patient had a partial response for 10 months and two patient had stable disease for 3.5 and 6.2 months, respectively	II	Completed
NCT06219174 (ClinicalTrials, 2024b)	DFMO plus pembrolizumab	This study evaluates the safety and clinical activity of Difluoromethylornithine (DFMO), an inhibitor of ornithine decarboxylase, as an immunotherapeutic target, in combination with pembrolizumab, a PD-1 monoclonal antibody, in advanced/metastatic NSCLC with *LKB1* mutations	N/A	I/II	recruiting
NCT06331650 (ClinicalTrials, 2024a)	carbognilumab plus chemotherapy	This study evaluates the safety and clinical activity of carbognilumab (DB16680), a PD-1 and CTLA-4 bispecific antibody, in combination with standard chemotherapy in advanced or postoperative recurrent NSCLC with *LKB1* mutations	N/A	II	recruiting
NCT03334617 (Besse et al., 2024)	durvalumab plus targeted therapy	This study evaluates the efficacy, safety and tolerability of durvalumab, a PD-L1 monoclonal antibody, in combination with targeted anticancer agents in refractory NSCLC. Patients with *LKB1* mutations were enrolled to receive durvalumab plus the PARP inhibitor olaparib	The objective response rate with durvalumab–olaparib was 4.8% in LKB1 biomarker matched cohort, suggesting limited vulnerability from targeting this mutation.	II	active, not recruiting

Hippo signaling is another critical pathway that mediates the effect of LKB1 on oncogenic growth control. This pathway is highly conserved in mammals where, canonically, the kinase MST1/2 phosphorylates LATS1/2 kinases, which in turn phosphorylate the transcriptional co-activator YAP, leading to its cytosolic retention and subsequent degradation. In the absence of phosphorylation, YAP translocates to the nucleus where it binds to TEAD (TEA-domain transcription factor) to activate a transcriptional program that drives tumor cell proliferation and growth. LKB1 promotes YAP phosphorylation through MARK, AMPK or TSSK1B, leading to YAP nuclear exclusion and proteasomal degradation and thus inactivation of its transcriptional activity (Figure 2). Loss of LKB1 in *KRAS* mutant lung cancer cells leads to YAP-mediated transcriptional activation, through regulation of localization of SCRIB, a scaffold protein involved in cell polarization, and Hippo kinases MST and LATS activity (Lenahan et al., 2023; Mohseni et al., 2014). Three LKB1 substrates, MARK1, 3 and 4, have also been found to control YAP-dependent transcription by complexing with LKB1, MST1, LATS1 and SCRIB (Mohseni et al., 2014). The LKB1-AMPK or LKB1-TSSK1B signaling pathways can phosphorylate YAP via LATS1/2 or directly phosphorylate YAP at a distinct site (Kim et al., 2024; Mo et al., 2015; Wang et al., 2015). Loss of YAP completely inhibited LKB1 deficient lung adenocarcinoma growth *in vivo*, indicating YAP as the critical mediator of oncogenic effects of LKB1 inactivation and YAP antagonism may represent a therapeutic approach for LKB1 mutant tumors.

SIKs are also reported to mediate the oncogenic effects of LKB1 inactivation in lung cancer. Knock-out of *SIK1* and *SIK3* increased tumor growth in a mouse model of oncogenic KRAS-driven lung cancer (Hollstein et al., 2019). Aberrant activation of CREB (CAMP responsive element binding protein) transcriptional activity through inactivation of SIK-CRTC (CREB regulated transcription coactivator) signaling underlines the aggressive phenotypes of LKB1-mutant lung cancer (Murray et al., 2019). LKB1 inactivation impairs SIKs phosphorylation of CRTCs, which leads to translocation of unphosphorylated CRTC into nucleus and activation of CREB-mediated transcription programs that promote cancer cell proliferation.

The tumor suppressor role of LKB1 is also linked to its effect on cell differentiation. In an oncogenic KRAS-driven mouse model of lung cancer where inactivation of LKB1 increases lung tumor burden, restoration of LKB1 activated the transcriptional program driven by CEBP (CCAAT enhancer binding protein), reinstated alveolar type II cell-like differentiation and consequently impeded proliferation and growth of lung tumors (Murray et al., 2022). Disruption of CEBP-driven lineage-specific transcriptional program following LKB1 inactivation led to loss of differentiated state and reversion to a progenitor-like state, which underscores the tumor suppressor role of LKB1 in lung cancer.

Beyond the effects on tumor growth, loss of LKB1 confers an invasive phenotype in various genetically engineered mouse models of cancer (Li et al., 2015). The process of cancer metastasis involves alterations in cell polarization and motility, cell detachment, and escape from anoikis, a programmed cell death induced by detachment from extracellular matrix. LKB1 regulates all these steps through AMPK-related proteins. LKB1 regulates cell motility through AMPK-mTOR-CREB (Pencik et al., 2023), cell polarization and microtubule organization through MARKs (McDonald, 2014), cell adhesion through FAK (focal adhesion kinase) (Kline et al., 2013) or NUAK (Zagórska et al., 2010) and anoikis via SIK (Cheng et al., 2009). A study of LKB1-dependent control of metastatic potential by Goodwin *et al.* defined a signaling pathway through MARK to regulate the expression level of SNAIL1, a critical transcriptional factor involved in induction of epithelial to mesenchymal transition (Goodwin et al., 2014). They showed that a scaffolding protein DIXDC1 (DIX domain containing 1) localizes to focal adhesions upon phosphorylation by MARK1. Loss of DIXDC1 results in upregulation of SNAIL1 in a FAK-dependent manner, leading to metastasis in mice (Goodwin et al., 2014). Taken together, LKB1 as a master kinase regulates cell growth and motility through complex signaling networks and its inactivation in cancer cells leads to a proliferative and metastatic phenotype (Figure 2). In the LKB1-driven signaling network, the AMPK-mTOR pathway is the most critical to target in LKB1-mutant tumors, especially due to the availability of clinically approved therapies.

### Metabolic reprogramming

**FIGURE 3:**
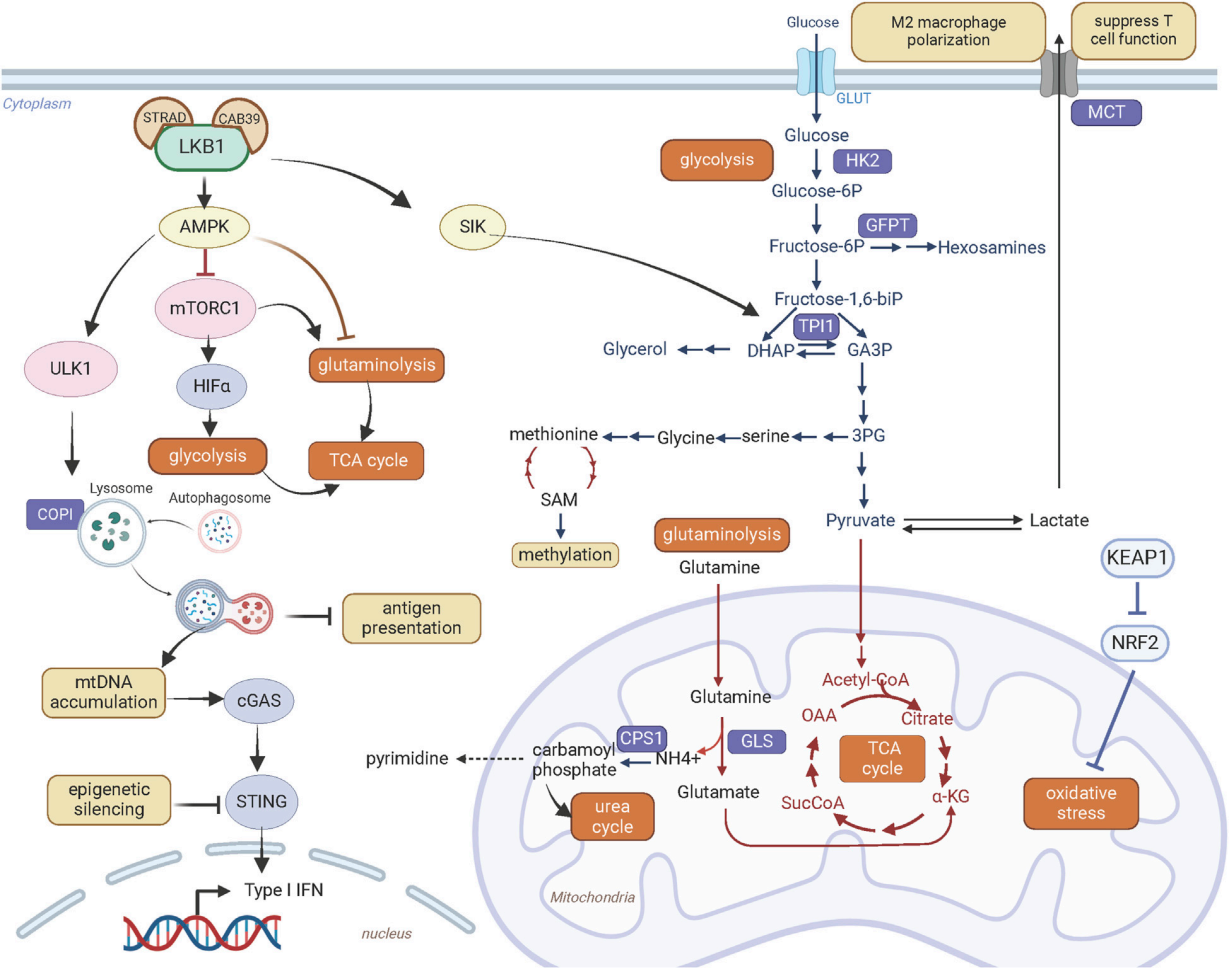
LKB1 promotes metabolic reprogramming and immune modulation. Created with BioRender.com.

In addition to regulating transcriptional and translational activity, LKB1 plays a crucial role in controlling metabolic activity. Cancer cells primarily depend on aerobic glycolysis to produce ATP and metabolic intermediates to support their proliferation and growth (Figure 3). This is characterized by increased lactate production in the presence of abundant oxygen, known as the “Warburg effect”. Loss of LKB1 promotes a metabolic switch to glycolysis (Dupuy et al., 2013; Faubert et al., 2014; Li and Zhu, 2020). This metabolic reprogramming in LKB1-deficent cells is shown to be dependent on HIF-1α activation by mTORC1, at least in part through AMPK signaling (Faubert et al., 2014). In addition, loss of LKB1 in human papillomavirus-positive cervical cancer cells enhanced glycolysis by increasing the expression of HK2 (hexokinase 2), an enzyme that catalyzes the first step of glucose metabolism by phosphorylating glucose to glucose 6-phosphate (Zeng et al., 2017).

The glycolysis pathway is linked to serine-glycine-one carbon pathway (Figure 3). The *de novo* synthesis of serine occurs through oxidation of the glycolysis intermediate 3-phosphoglycerate. LKB1 inactivation in *KRAS* mutant lung cancer cells enhances glycolysis and serine biosynthesis and protects cell survival from serine deprivation through deregulated AMPK-mTORC1 signaling (Kottakis et al., 2016). Elevated serine metabolism can increase abundance of SAM (S-adenosyl methionine) through methionine metabolic pathway. SAM transfers methyl groups to DNA, RNA, proteins or lipids catalyzed by DNA methyltransferases. The increased SAM content and DNA methylation with upregulated DNA methyltransferases suggest metabolic reprogramming coupled with epigenetic alterations contribute to oncogenic effects of LKB1 deficiency in *KRAS* mutant pancreatic cancer cells. Furthermore, these epigenetic alterations provide transcriptional plasticity of LKB1 mutant cells under metabolic stress and confer sensitivity to the DNA methyltransferase inhibitor Decitabine (Kottakis et al., 2016).

Loss of LKB1 is associated with a more aggressive tumor growth in genetically engineered mouse models of lung cancer harboring both *Tp53* and *Kras* mutations. Interestingly, these three oncogenic mutations rarely co-occur in human lung cancer. A recent study by Stein *et al.* explained this species discrepancy from the effect of LKB1 on energy metabolism (Stein et al., 2023). They found that loss of *LKB1* in *KRAS* and *TP53* co-mutant human lung cancer cells decreased phosphorylation of TPI1 (Triosephosphate isomerase 1), a metabolic enzyme that catalyze the interconversion between glyceraldehyde-3-phosphate (GA3P) and dihydroxy-acetone phosphate (DHAP), at serine 21 through SIK (Figure 3). Decrease of TPI1 activity following LKB1 loss shifts energy balance away from energy production via glycolysis and towards glycerol lipid production and energy conservation. This metabolic alteration may exaggerate metabolic stresses experienced during tumorigenesis and is disadvantageous for cell proliferation and growth. In mouse lung cancer cells, instead, there is a substitution of an oxidizable cysteine for serine at residue 21 of TPI1. This mutation abrogates LKB1-SIK-mediated phosphorylation. Consequently, in the absence of TPI1-mediated energy regulation, loss of LKB1 improved the efficiency of energy production through glycolysis, underlying the synergy of these three oncogenic mutations in mouse lung tumor growth. Conversely, LKB1 deficiency in human lung cancer cells with *TP53* and *KRAS* co-mutations have inferior metabolic state for cancer cell fitness through regulation of TPI1, providing a mechanistic explanation for the rare co-mutations of *LKB1*/*KRAS*/*TP53* in human lung cancer.

Beside glucose, glutamine through glutaminolysis provides carbon source for synthesis of metabolites and energy production (Figure 3). This metabolic pathway converts glutamine by glutaminase to glutamate which is then catabolized to α-ketoglutarate via glutamate dehydrogenase 1 to fuel tricarboxylic acid cycle. LKB1 inactivation in *KRAS* mutant lung cancer cells promotes glutaminolysis and facilitates the formation of glutamine-derived succinate, fumarate and malate in the later stages of TCA cycle, thus promoting mitochondrial oxidative phosphorylation (Caiola et al., 2018). Elevated glutamine catabolism in *LKB1* and *KRAS* co-mutant cells leads to accumulation of ammonia. CPS1 (Carbamoyl-phosphate synthase 1) uses ammonia and bicarbonate to produce carbamoyl phosphate in the mitochondria. Carbamoyl phosphate either enters urea cycle to convert highly toxic ammonia to urea for excretion or participates in metabolic pathways for the synthesis of pyrimidines (Figure 3). An increase of CPS1 expression and urea cycle metabolites in *KRAS* and *LKB1* co-mutant lung cancer cells increases pyrimidine synthesis and benefits for tumor growth (Kim et al., 2017).

Moreover, elevated mitochondrial oxidative phosphorylation promotes reactive oxygen species (ROS) production. Tumors bearing *LKB1* deletion have high levels of ROS, which provoke damages to cellular organelles and processes, and ultimately impair cellular fitness. In NSCLC, *LKB1* is often co-mutated with *KEAP1* deletion, defining an additional subgroup (Galan-Cobo et al., 2019). Loss of KEAP1 leads to accumulation of NRF2, a master antioxidant transcription factor that protects cells against oxidative stress (Figure 3). Galan-Cobo et al. (2019) characterized the metabolic phenotypes of *KRAS*/*LKB1*/*KEAP1* co-mutant lung cancer. They defined addiction to glutamine metabolism in this subgroup of lung cancer confered high sensitivity to the glutaminase inhibitor CB-839 *in vitro* and *in vivo*.

However, with an increase in metabolic capacity, metabolic plasticity is reduced. This render *LKB1* mutant cancer cells sensitive to metabolic stress caused by nutrient deprivation (Caiola et al., 2018). The dependence on glucose and glutamine metabolism thus can be leveraged to treat tumors with LKB1 mutations. The biguanide metformin is widely used to treat diabetes due to its ability to lower glucose, insulin, and IGF1 plasma levels and attenuate insulin resistance by increasing glucose uptake (Nasri and Rafieian-Kopaei, 2014). Metformin has been repurposed for anti-cancer treatments. In LKB1-deficient lung cancer cells, metformin induces apoptosis by inhibiting HIF-1α activity and promoting degradation of pro-survival proteins (Faubert et al., 2014; Huang et al., 2020; Luo et al., 2019). It also prevents resistance to cisplatin in *LKB1* and *KRAS* co-mutated lung cancer cells by targeting tumor-initiating cells (Moro et al., 2018). A Phase II clinical trial, FAME, is currently underway to evaluate the effectiveness of metformin alone or in combination with a fasting-mimicking diet to improve standard chemotherapy outcomes for patients with *LKB1*-mutant lung adenocarcinoma (Vernieri et al., 2019). In addition, elevated glutamine metabolism supports targeting glutaminase as an approach to treat *LKB1* mutant cancers (Sitthideatphaiboon et al., 2021). However, Telaglenastat (CB-839), a glutaminase inhibitor did not show clinical benefit in LKB1 mutant tumors either as a monotherapy or in combination with immunotherapy and chemotherapy (Table 1) (Skoulidis et al., 2020).

In addition, new metabolic vulnerabilities that have been identified through genetic approaches can be exploited to target LKB1 mutant tumors. Kim et al. (2013) identified that lung cancer cells with *KRAS* and *LKB1* co-mutations rely on COPI (coatomer complex I)-dependent lysosome acidification, an essential step for lysosome maturation (Figure 3). As lysosomes degrade macromolecules and supply metabolic intermediates for TCA cycle, lysosomal function is coupled to mitochondrial health. Genetically or pharmacologically targeting COPI complex causes mitochondrial dysfunction and consequently cell death (Kim et al., 2013). Liu et al. (2013) also identified that DTYMK (deoxythymidylate kinase) is synthetically lethal with LKB1 deficiency in *KRAS* mutant lung cancer cells. DTYMK catalyzes the phosphorylation of thymidine monophosphate (dTMP) to thymidine diphosphate (dTDP) and thus contributes to dTTP biosynthesis. Its inhibition reduces the dTDP pool and leads to an accumulation of dUTP which is incorporated in DNA, causing DNA damage. *LKB1*-deleted cells were more dependent on the dTTP synthesis pathway due to the lower expression of DTYMK, and this defect in nucleotide metabolism renders LKB1 deficient cells sensitive to the inhibition of DTYMK. In addition, *KRAS*/*LKB1* co-mutated cells were found to depend on an enzyme involved in the hexosamine biosynthesis, glutamine-fructose-6-phosphate transaminase 2 (GFPT2) (Kim et al., 2020). GFPT2 inhibition selectively impedes the tumor growth of this molecular subtype.

Overall, LKB1 is a master regulator of energy production, mainly through AMPK (Figure 3). LKB1 deficiency in cancer cells promotes metabolic reprogramming to support tumor growth and metastasis. These metabolic dependencies become potential vulnerabilities in *LKB1* mutant tumors and provides new therapeutic intervention opportunities to develop personalized treatment for cancers with *LKB1* mutations.

### DNA damages and genomic instability

The constant insults from endogenous (e.g., nucleotide metabolism) and exogenous sources (e.g., UV or ionizing radiation, chemical or biological genotoxins) cause DNA damages. The DNA damage response (DDR) maintains genome stability and cellular viability, via a complex system of signaling pathways that include damage sensors, signal transducers and effectors of cell cycle checkpoints and DNA repair (Groelly et al., 2023). Defective DDR leads to genome instability, a hallmark of cancer, that predisposes cells to malignant transformation and progression driven by secondary events of activation of oncogenes and loss of tumor suppressor genes. LKB1 has been suggested to play a crucial role in homologous recombination (HR) repair in response to DNA double-strand breaks (DSBs). LKB1 inactivation hinders HR repair in response to irradiation and chemotherapeutic agents (Deng et al., 2021; Wang et al., 2016). Consequently, LKB1 deficient tumors exhibit high levels of genomic instability. In a KRAS-driven NSCLC mouse model, loss of *Lkb1* increases tumor mutational burden compared with *Tp53* mutation. Specifically, *Lkb1* deficiency leads to an increase in the number of coding insertion-deletion and non-synonymous single nucleotide variations, reinforcing its important role in preserving genome stability (Wang et al., 2016).

LKB1 has been demonstrated to be a target of the protein kinase ATM, a central component of DDR that orchestrates responses to DSBs and stalled DNA replication forks (Sapkota et al., 2002; Wang et al., 2016). Phosphorylation of LKB1 at Thr363 by ATM was observed as early as 15 min after irradiation, supporting that it is a specific response to DNA DSBs (Sapkota et al., 2002). LKB1 forms DNA damage-induced nuclear foci in a complex with other DDR proteins including γ-H2AX, BRCA1 and ATM and facilitates the recruitment of RAD51 to chromatin for HR repair, providing molecular basis for the involvement of LKB1 in DNA repair (Wang et al., 2016). LKB1 deficiency causes DNA repair defects and confers sensitivity to cisplatin and poly-ADP ribose polymerase (PARP) inhibitors (Wang et al., 2016). As part of DDR, the mitotic checkpoint acts as a key surveillance mechanism of genome integrity by stalling cell division till DNA repair is completed. AZD1775, an inhibitor of the G2-M cell cycle checkpoint protein WEE1, in combination with cisplatin or radiation enhanced DNA damages through bypass of the G2-M checkpoint and improved survival of mice with *Kras* and *Lkb1* co-mutant lung cancer (Richer et al., 2017). Similarly, CHK1 inhibitor AZD7762 was reported to synergize with the DNA-damaging drug gemcitabine in reducing cell viability and suppressing tumor growth of LKB1 deficient lung cancer (Liu et al., 2017). The effects of LKB1 on DDR and genome stability are thought to attribute to nucleus-localized LKB1. But the contribution of cytoplasmic LKB1 through metabolic and growth control cannot be excluded and warrants further investigation. More importantly, evaluation of the association of LKB1 mutation status with chemotherapy response will raise the potential of LKB1 as a therapeutic biomarker in cancer treatment.

### Immune modulations

Recent studies have shown that, in addition to its direct effects on tumor cells, LKB1 plays an important role in regulating the tumor microenvironment. LKB1 inactivation in lung adenocarcinoma is associated with an immunosuppressive phenotype. It is characterized by suppression of T cell infiltration, particularly the number and function of effector CD8^+^ T cells (Koyama et al., 2016; Skoulidis et al., 2015), inhibition of PD-L1 expression and IFN-γ signaling, increase of recruitment of granulocytic myeloid-derived suppressor cells (Li et al., 2021) and secretion of immunosuppressive cytokines such as IL-6 (Koyama et al., 2016). Loss of *LKB1* in *KRAS*-mutant lung adenocarcinoma leads to a distinct immune profile compared to *TP53* null tumors (Biton et al., 2018; Skoulidis et al., 2015). Tumors with *KRAS* and *TP53* co-mutations exhibit an adaptive immune response with immune activation evidenced by enhanced T-cell infiltration and upregulation of expression of cell intrinsic immune checkpoint signals such as PD-1/PD-L1. In contrast, tumors with *KRAS*/*LKB1* co-mutations show a cold immune microenvironment with lack of immune system engagement. This poor immune surveillance underlies the low sensitivity of immunotherapies in lung adenocarcinoma patients with LKB1 deficiency. *LKB1* mutation status is associated with intrinsic resistance to immunotherapy checkpoint blockade (ICB) in *KRAS*-mutant non-small cell lung cancer (NSCLC) (Koyama et al., 2016; Skoulidis et al., 2018). Several retrospective studies have evaluated the predictive potential of LKB1 status on ICB response but the results remain inconclusive (Pons-Tostivint et al., 2021).

The immunosuppressive phenotype of LKB1 deficient lung cancer is associated with the regulatory role of LKB1 in metabolism. As discussed above, loss of LKB1 leads to a metabolic switch to aerobic glycolysis and increase of lactate secretion (Figure 3). Upregulation of MCT4 (also known as solute carrier family 16 member 4 or SLC16A4), a major lactate transporter, contributes to the immunosuppressive phenotype including M2 macrophage polarization and T cell hypofunction observed in a *Lkb1*-deficient *Kras*-driven lung cancer mouse model (Qian et al., 2023). The *in vivo* evidence supports that suppression of glycolysis by depletion of MCT4 transporter or blocking lactate receptor GPR81 can abrogate M2 macrophage polarization, partially restore T cell function, and overcome PD-1 inhibitor resistance. Further studies are required to establish the clinical relevance of lactate metabolism in immunotherapy resistance of human *LKB1* mutant lung tumors.

Restricted antigen presentation to MHC complexes has been attributed to low T cell infiltration in *LKB1*and *KRAS* co-mutant tumors. Deng *et al* reported *LKB1* loss increased autophagic flux and reduced proteasomal degradation of antigen peptides, thereby suppressing antigen presentation (Deng et al., 2021). Inhibiting autophagy by targeting ULK1 restored antigen presentation through enhancing immunoproteasome activity and synergized with PD-1 inhibition to promote anti-tumor immunity.

STING is a key regulator of the innate immune response by linking detection of aberrant cytoplasmic double-strand DNA by cGAS to activation of innate immune signaling that facilitate T-cell recruitment (Figure 3). LKB1 inactivation in *KRAS* mutant lung cancer cells causes cytoplasmic accumulation of mitochondrial DNA due to defects in autophagy and mitochondrial dysfunction, which triggers activation of STING signaling. However, STING expression is suppressed in *LKB1* mutant cells through epigenetic silencing via the methyltransferases EZH2 and DNMT, consequently disrupting cytoplasmic mitochondrial DNA sensing (Kitajima et al., 2019). Reinduction of LKB1 restored STING expression and rescued chemokine production that promote T-cell recruitment. To develop therapeutic approaches to restore and activate STING signaling and enhance immunogenicity in *KRAS* and *LKB1* co-mutant lung cancer, Kitajima et al. (2022) performed a drug screen and identified inhibition of MPS1 to activate cGAS-STING pathway signaling. MPS1 (also known as TTK protein kinase, TTK) is a critical regulator of the mitotic spindle assembly checkpoint. Abrogation of the mitotic spindle assembly checkpoint by MPS1 inhibition disrupts chromosome segregation, increases chromosome instability and generates micronuclei in cytoplasm, which are recognized by the cGAS-STING pathway. Furthermore, they designed a sequential treatment of an epigenetic inhibitor decitabine to restore STING expression followed by pulse treatment with the MPS1 inhibitor BAY-1217389 to activate the STING pathway. This sequential combination therapy enhanced T cell recruitment and improved sensitivity to PD-1 blockade in mice harboring *Kras* and *Lkb1* mutated lung cancer (Kitajima et al., 2022).

Collectively, loss of LKB1 not only alters intracellular signaling and cellular functions but also modifies the extracellular microenvironment, enabling cancer cells to evade cancer immunosurveillance thus promoting intrinsic resistance to immunotherapy. Several new ICBs in combination with targeted therapies or chemotherapies are currently tested in clinic for tumors with LKB1 mutations (Table 1). Further understanding of the impacts of LKB1 deficiency on cancer cell immunogenicity and tumor immune infiltration will facilitate development of new strategies to increase the clinical efficacy of immunotherapy in patients with *LKB1* mutated cancers.

## LKB1 in ovarian cancer

### Ovarian cancer

Annually 300,000 women throughout the world are diagnosed with ovarian cancer. Sadly, late-stage diagnosis occurs in 58% of cases with the 5-year survival rate of only 30% post-metastasis, in contrast to 92% when the disease is detected early, which strengthens the importance of timely screening and early intervention (Arora et al., 2024). Despite its prevalence and impact, early diagnosis remains challenging due to vague clinical symptoms and the lack of preventative screening methods (Arora et al., 2024).

Ovarian cancer comprises four subtypes: epithelial, germ cell, small cell carcinomas, and sex-cord stromal tumors. Epithelial ovarian cancer (EOC), the most prevalent (90% of cases), have five histological types which are categorized as type I or type II(Hollis, 2023). Type I tumors, including low-grade serous ovarian cancer, endometrioid, clear-cell, and mucinous carcinomas, are generally confined to the ovary and not invasive. These tumors are often identified at an early stage with good prognosis. In contrast, type II tumors, notably high-grade serous ovarian cancer (HGSOC), which accounts for 70%–80% of ovarian cancer cases, are highly proliferative and aggressive with high chromosomal instability (Vang et al., 2009). The most common mutations in HGSOC are *TP53* (90%), *BRCA1/2* and other HR genes (50%) and genes involved in PI3K and RAS pathways (45%) (Yan et al., 2017).

Owing to the difficulty in early diagnosis and lack of preventative screening, the site and cell of origin of serous carcinoma are still under debate. Both ovarian surface epithelium (OSE) and fallopian tube epithelial (FTE) cells have been considered as the origin for serous ovarian epithelial cancer. Nevertheless, recent genomic studies support HGSOC is mostly originated from the epithelium of distal fallopian tube where a peculiar lesion called serous tubal intra-epithelial carcinoma (STIC) forms as a precursor of HGSOC (Kuhn et al., 2016; Kurman and Shih, 2016; Labidi-Galy et al., 2017).

The standard treatment for ovarian cancer includes surgical debulking, systemic chemotherapy and targeted therapy (Arora et al., 2024). However, recurrence following surgery and chemotherapy is common. 90% of HGSOC acquire resistance to chemotherapy within 2-3 years. HR-deficiency is a key determinant of sensitivity to chemotherapy and PARP inhibitors. The introduction of PARP inhibitors like olaparib, rucaparib, and niraparib, as maintenance therapy has dramatically improved the progression-free survival of HR-deficient HGSOC. However, 30%–50% of cases that initially respond to PARP inhibitors develop resistance leading to incurable disease (Wakefield et al., 2019). Mechanisms of resistance to PARP inhibitor include restoration of HR activity mostly through reversion mutations, stabilization of stalled DNA replication forks, metabolic alterations, and increased drug efflux (Burdett et al., 2023; Wakefield et al., 2019). No effective therapeutic options exist for resistant HGSOC, making the search for new therapies a critical unmet clinical need.

### LKB1 expression and mutations in ovarian cancer

**FIGURE 4:**
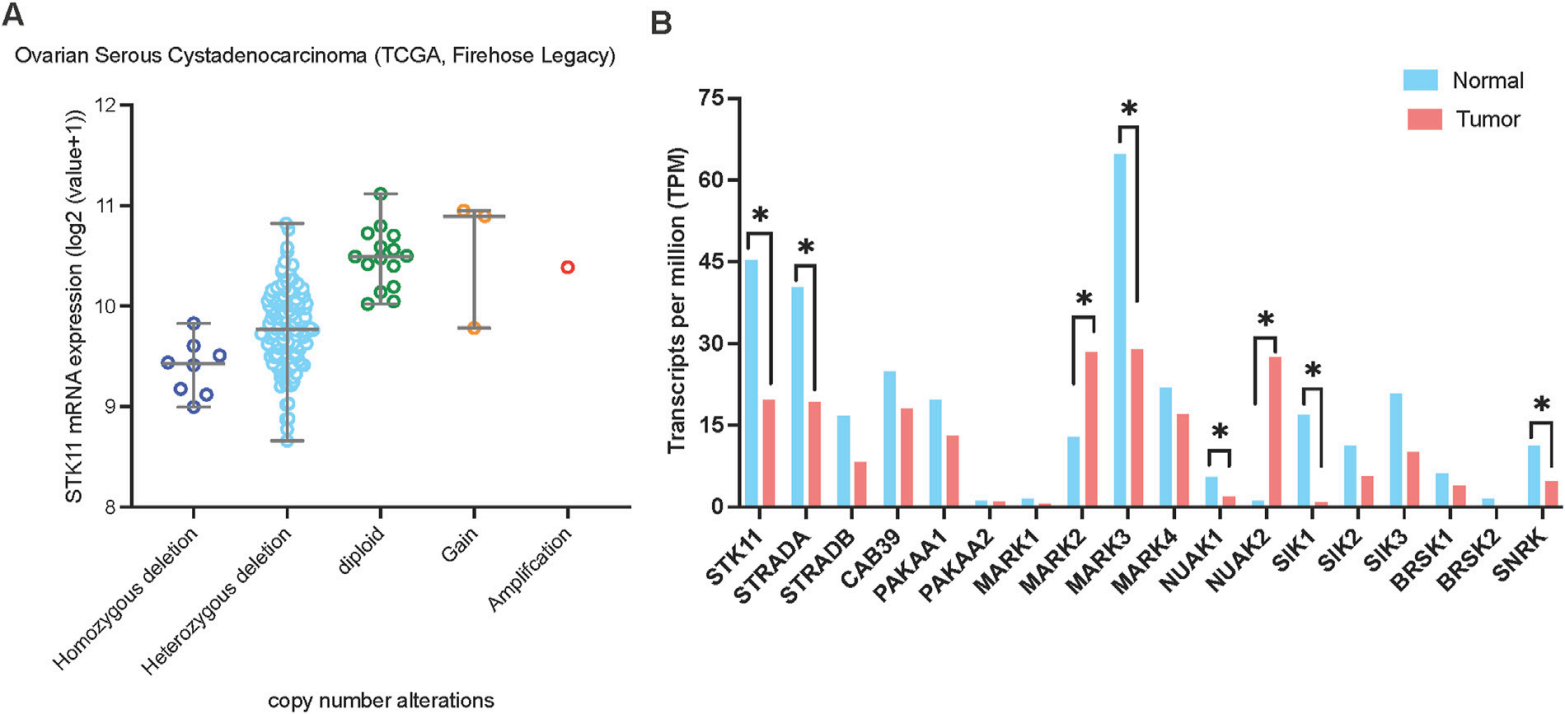
*LKB1* copy number alterations and mRNA expression in ovarian cancer. **(A)** *LKB1* mRNA expression levels in ovarian serous cystadenocarcinoma correlate with *LKB1* copy number alterations. The data was assessed from cBioPortal (www.cbioportal.org). **(B)** The mRNA expression level of the genes involved in LKB1 signaling in ovarian cancer. The data was assessed from GEPIA on 5th June 2024.

To characterize *LKB1* genetic alteration in ovarian cancer, we analyzed a TCGA ovarian serous cystadenocarcinoma dataset (N = 182) and identified homozygous and heterozygous gene deletions in 4.4% (N = 8) and 85.2% (N = 155) of samples, respectively (Figure 4A). Similarly, genomic analysis of a small cohort of 75 HGSOC cases by Tanwar et al. (2014) identified copy number loss in 31% (23/75), allelic imbalance in 64% (48/75) and copy number gain in only 1/75 of samples. In a cohort study of 62 Chinese patients with epithelial ovarian cancer, deleterious germline LKB1 mutations have been found in 5.3% (Li et al., 2019).

Moreover, two studies examined LKB1 protein expression in human epithelial ovarian cancer. George *et al* reported that LKB1 expression was predominantly reduced in 71% of HGSOC (213/298), compared to 47% of clear-cell carcinoma (15/32), 29% of mucinous carcinoma (8/28) and 22% of endometrioid carcinoma (6/27), assessed by immunohistochemical analysis of a tissue microarray (George et al., 2016). In the HGSOC cohort, there was no significant correlation between LKB1 protein expression and progression-free survival or overall survival. Notably, only a third of the samples with reduced LKB1 protein expression had a gene copy number deletion, suggesting additional mechanisms of LKB1 silencing exist in HGSOC. They also examined 15 cases of STIC, the precursor of HGSOC for LKB1 expression. A significant decrease of LKB1 protein expression was observed in 13/15 cases, suggesting loss of LKB1 activity as an early event in HGSOC development. The other study of LKB1 protein expression conducted by Tanwar *et al* reported that 54% of HGSOC samples (N = 92) showed absence of LKB1 expression (Tanwar et al., 2014). These studies suggest that the loss or reduced expression of LKB1 is a common alteration in HGSOC.

### LKB1 in early serous ovarian tumorigenesis

To understand the functional impacts of LKB1 loss in the aetiology of serous ovarian cancer, Tanwar et al. (2014) conditionally deleted *Lkb1* in mouse OSE cells. This led to abnormal papillary growth and widespread shedding of surface epithelial cells. Simultaneous deletion of *Pten* and *Lkb1* led to the development of high-grade papillary serous carcinomas with increased mTOR activity. In a complementary approach, George et al. (2016) used a series of primary FTE cell lines harboring dominant negative form of TP53 (TP53-R175H) or an immortalized FTE cell line with three genetic modification including expressing TERT, a dominant negative TP53 and Rb loss, to study the role of LKB1 in serous tumorigenesis. Interestingly, loss of LKB1 in *TP53* mutant primary and immortal FTE cells led to premature cellular senescence and resulted in a G2/M cell cycle arrest. Overexpression of cyclin E1 bypassed LKB1-induced senescence, re-initiated cell proliferation and promoted anchorage-independent growth of FTE cells. Consistent with its role in control cell polarity, defect of apical to basal polarity was also observed in LKB1 deficient primary FTE cells and a significant destabilized epithelial integrity in *LKB1*-*TP53* co-mutated cells. These findings reinforce the concept that the loss of LKB1 is an early genetic alteration that co-operates with other oncogenic events, to affect cell polarity and differentiation and promote oncogenic transformation.

To test this concept, we examined a TCGA data set (Table 2) and identified 5.1% (16/311) of serous ovarian cancer with *LKB1* deletion. *LKB1* deletion co-occurs with common HGSOC genetic alterations, particularly with *CCNE1* mutations, but are not detected in tumors with *BRCA1* or *BRCA2* mutations. Further analysis of clinical genomic data will provide more understanding of synergy of LKB1 deficiency and other oncogenes/tumor suppressors in ovarian tumorigenesis.

**TABLE 2:** Frequency of LKB1 deep deletion co-occurring with common mutations in HGSOC. Analysis of 311 ovarian cancer serous cystadenocarcinoma in TCGA dataset via http://www.cbioportal.org (access date 27th September 2024). Statistical analysis was performed using Fisher’s exact test.

Mutant gene	Number of patients	Number of patients with *LKB1* co-mutations	% Occurrence of *LKB1* co-mutation	p-value	95% CI	Odds ratio
*CCNE1*	67	6	8.96	0.12	0.66, 7.29	2.29
*RB1*	39	2	5.13	1	0.11, 4.62	1
*TP53*	275	14	5.09	1	0.20, 8.61	0.91
*PTEN*	21	1	4.76	1	0.02, 6.59	0.92
*MYC*	128	5	3.91	0.45	0.17, 2.05	0.64
*NF1*	37	1	2.70	0.70	0.01, 3.31	0.48
*BRCA1*	20	0	0	0.61	0.00, 3.89	0
*BRCA2*	22	0	0	0.61	0.00, 3.49	0

### LKB1 in ovarian cancer progression

In contrast to the established tumor suppressor role in serous ovarian cancer initiation, there is no consensus on LKB1’s role in ovarian cancer progression. The Shepherd group exploited a three-dimensional model system of spheroid formed by spontaneous cell aggregation in suspension, to study the role of LKB1 during metastatic progression of EOC (Tomas and Shepherd, 2023). They reported that depletion of *LKB1* by siRNA or CRISPR-Cas9 did not affect proliferation of EOC cells in adherent 2D culture conditions. However, LKB1 depletion significantly impaired anchorage-independent growth of EOC cells and reduced viability of the spheroids, implicating LKB1 loss abrogates EOC metastatic potential (Buensuceso et al., 2020; Peart et al., 2015). They further injected EOC cells directly into the peritoneal space of immunodeficient mice and demonstrated that loss of LKB1 improved survival, reduced tumor burden and hindered disease spreading in this orthotopic mouse xenograft model of metastasis (Buensuceso et al., 2020). Their studies suggest that functional LKB1 signaling is required to maintain dormancy of EOC cells in spheroids growth and protect from anoikis during the process of dissemination to other organs.

Studies from the Shepherd group also demonstrated that depletion of AMPK by siRNA in EOC spheroids had no effect on cell viability, suggesting the protective effect of LKB1 in EOC spheroids is independent of AMKP signaling. One substrate of LKB1, NUAK1 has been reported to promote EOC spheroid formation through fibronectin expression (Fritz et al., 2020) and its expression is associated with poor prognosis in ovarian cancer (Phippen et al., 2016). Loss of LKB1 significantly decreased NUAK1 expression in adherent EOC cells and spheroids (Fritz et al., 2020), suggesting that NUAK1 may mediate the biological effects of LKB1 on spheroid formation, anoikis-resistance and metastatic potential. Loss of LKB1 or NUAK1 in EOC spheroid cells leads to accumulation of reactive oxygen species and subsequently induces activation of NF-kB signaling as an adaptive response to oxidative stress (Buensuceso et al., 2022). Taken together, their studies suggest that an intact LKB1 signaling is required for EOC metastasis.

To gain further insights into the functional impact of LKB1 on ovarian cancer progression, we analyzed the mRNA expression levels of *LKB1* and its major downstream 13 kinase in ovarian tumor tissues compared with normal tissues using ovarian cancer datasets from TCGA and GTEx projects through GEPIA (Tang et al., 2017) (Figure 4B). Most genes showed downregulation at the mRNA level in ovarian tumor tissues except *MARK2* and *NUAK2*. Overall, the data suggests that LKB1 signaling is suppressed in ovarian tumors. Furthermore, a recent study reported that one LKB1 substrate, MARK3 has a tumor suppressor role in HGSOC (Machino et al., 2022). Activation of MARK3 under metabolic stress phosphorylates CDC25B and induces cell cycle G2/M phase arrest (Figure 2). Depletion of LKB1 abrogated MARK3 activation, which may contribute to increased cell proliferation.

Collectively, the contradictory data of the role of LKB1 in ovarian cancer cell survival and invasiveness suggests its effects could be genetic context-dependent through interaction with other oncogenic signaling. It is also possible that the requirement for LKB1 signaling is varied at different stages of cancer progression from primary tumor growth, distal metastasis to tumor recurrence and adjusted by different metabolic stresses and microenvironment. This complexity thus confers the heterogenous phenotypes of LKB1 deficient ovarian tumors.

## Conclusion and perspectives

LKB1 has been identified as a gene responsible for PJS, a rare autosomal dominant disease with an increased predisposition to malignant tumors in multiple tissues. While *Lkb1* knockout mice are predisposed to developing cancer, LKB1 loss alone is not sufficient to initiate tumorigenesis. Accumulating evidence supports that LKB1 loss might occur as a secondary oncogenic lesion that facilitate transformation by at least one constitutively active oncogene. As a tumor suppressor, LKB1 phosphorylates its target substrates and regulates their activities to exert its biological functions primarily impacting on cell growth, metabolism and polarity. While AMPK as the canonical downstream kinase regulated by LKB1 has been well studied, the roles of other target kinases in mediating LKB1 functional impacts in cancer biology are still less defined. Further characterization of these substrates is crucial to understand the multi-functions of LKB1.

Deletion of *LKB1* is a common genetic alteration in epithelial ovarian cancer. Studies in human FTE cell lines and mouse OSE cells suggest that loss of LKB1 co-occur with other oncogenic mutations including TP53 inactivation, overexpression of cyclin E1 or loss of *PTEN* during ovarian transformation despite lack of mechanistic investigation. The studies in an ovarian cancer spheroid model system, however, suggest that LKB1 function is required for anchorage-independent growth and metastasis potential in ovarian cancer cells. It should be noted that these ovarian cancer cell lines have heterogenous genomic characteristics and LKB1 deficiency may have distinct functional impacts via interactions with various oncogenic mutations. To advance our understanding of LKB1 signaling in ovarian cancer, particularly HGSOC, several areas warrant further investigations: (i) The evaluation of the prevalence of *LKB1* mutations, and co-occurring genomic alterations, as well as the impact on prognosis, response to therapy and survival outcomes will help define the molecular subtypes of *LKB1* mutant ovarian cancer. (ii) In addition to commercially available cell lines, HGSOC patient-derived cell lines may provide more clinically relevant *in vitro* models to study the biology of LKB1 (Pishas et al., 2021). (iii) Given LKB1 is a master kinase which primarily regulates cell metabolism and cell growth, deciphering signal transduction and metabolic alterations is required to determine the functional impact of LKB1 deficiency, in synergy with the common genetic mutations in ovarian cancer cells. (iv) Comprehensive profiling of the tumor microenvironment of *LKB1* mutant ovarian cancer using patient samples and syngeneic mouse ovarian cancer models may provide new mechanistic insight into the relative low response rate of EOC to immune therapy. Altogether, such studies will identify therapeutic vulnerabilities and enable developing personalized treatments for patients with *LKB1* mutant ovarian tumors.
